# Artificial light at night causes diapause inhibition and sex-specific life history changes in a moth

**DOI:** 10.1002/ece3.1090

**Published:** 2014-04-25

**Authors:** Koert G van Geffen, Roy H A van Grunsven, Jasper van Ruijven, Frank Berendse, Elmar M Veenendaal

**Affiliations:** 1 Nature Conservation and Plant Ecology Group, Wageningen UniversityDroevendaalsesteeg 3a, P.O. box 47, 6700 AA, Wageningen, the Netherlands; 2 Department of Animal Ecology, Netherlands Institute of Ecology (NIOO-KNAW)Droevendaalsesteeg 10, P.O. box 50, 6700 AB, Wageningen, the Netherlands

**Keywords:** Caterpillars, development time, diapause, light pollution, pupal mass, pupation

## Abstract

Rapidly increasing levels of light pollution subject nocturnal organisms to major alterations of their habitat, the ecological consequences of which are largely unknown. Moths are well-known to be attracted to light at night, but effects of light on other aspects of moth ecology, such as larval development and life-history, remain unknown. Such effects may have important consequences for fitness and thus for moth population sizes. To study the effects of artificial night lighting on development and life-history of moths, we experimentally subjected *Mamestra brassicae* (Noctuidae) caterpillars to low intensity green, white, red or no artificial light at night and determined their growth rate, maximum caterpillar mass, age at pupation, pupal mass and pupation duration. We found sex-specific effects of artificial light on caterpillar life-history, with male caterpillars subjected to green and white light reaching a lower maximum mass, pupating earlier and obtaining a lower pupal mass than male caterpillars under red light or in darkness. These effects can have major implications for fitness, but were absent in female caterpillars. Moreover, by the time that the first adult moth from the dark control treatment emerged from its pupa (after 110 days), about 85% of the moths that were under green light and 83% of the moths that were under white light had already emerged. These differences in pupation duration occurred in both sexes and were highly significant, and likely result from diapause inhibition by artificial night lighting. We conclude that low levels of nocturnal illumination can disrupt life-histories in moths and inhibit the initiation of pupal diapause. This may result in reduced fitness and increased mortality. The application of red light, instead of white or green light, might be an appropriate measure to mitigate negative artificial light effects on moth life history.

## Introduction

Over the past decades, increasing levels of artificial night lighting have led to large-scale alterations of night-scapes worldwide (Cinzano et al. 2001; Elvidge et al. 2001). Although there are remarkable differences in trends between countries and regions (Bennie et al. 2014), levels of artificial night lighting are currently still increasing annually with 6% on average (Hölker et al. 2010a). Although effects of artificial night lighting have been reported in diurnal species (Kempenaers et al. 2010; Titulaer et al. 2012), the ever-increasing levels of artificial night lighting may particularly affect nocturnal species, representing the majority of terrestrial fauna (Hölker et al. 2010b), as they are increasingly subjected to illumination of their habitat. Moths are a well-known example of organisms that are affected by artificial light at night, as they are strongly attracted to light, although significant differences exist among species and families (Van Langevelde et al. 2011; Truxa and Fiedler 2012; Somers-Yeates et al. 2013; Merckx and Slade 2014). Besides attraction, negative effects of artificial light at night on other aspects of moth ecology, such as survival, reproduction and development have been suggested (Frank 1988), but these have rarely been studied. For example, effects of artificial light on moth life history are unknown to date. In this respect, effects of artificial light on caterpillars is highly relevant as this is the life stage when important life history trade-offs, such as time to reach maturity and size at maturity, are faced. Unfavourable conditions during the larval stage are known to lead to lower caterpillar growth rates, advanced pupation and reduced pupal mass (Tammaru et al. 2000). This in turn can affect important fitness measures such as female egg production (Honěk 1993; Tammaru et al. 2002), mate preference (Gage 1998; Van Dongen 1998; Iyengar and Eisner 2002; Xu and Wang 2009), longevity (Shirai 1995; Tammaru et al. 2002) and flight ability (important for gene spread; Shirai 1993, 1995; Merckx and Van Dyck 2006). In addition, natural light conditions are often used by organisms as a source of information about circadian and seasonal timing (Gaston et al. 2013). In many moth species, caterpillars use day length as the main environmental cue for the decision to initiate pupal diapause, where short days trigger diapause (e.g., Adkisson 1966; Huang et al. 2005; Xiao et al. 2010), ensuring that late generation caterpillars or pupae overwinter in diapause so that the imago life stage is synchronised with favourable seasons (Adkisson 1966). Light at night can disturb the reliability of this cue (Gaston et al. 2013) by altering day length as perceived by caterpillars, which may lead to differences in timing or even inhibition of diapause.

In this study, we experimentally investigate the effects of various types of artificial night lighting, differing in spectral composition, on life history of *Mamestra brassicae* (L.) (Noctuidae). In Western Europe, this species has two generations per year of which the first generation pupates in summer and the second generation overwinters as a diapausing pupa (Steiner and Elbert 1998; Waring and Townsend 2003). As in many other moth species, caterpillars of *M. brassicae* are nocturnal.

We hypothesize that artificial light at night lengthens perceived day length, leading to a lower caterpillar growth rate, postponed pupation and reduced pupal mass. Furthermore, we expect that artificial light would lead to shorter duration of the pupal because it might interfere with day-length as a cue for diapause initiation and thus inhibit induction of diapause. Because caterpillar eyes (stemmata) are most sensitive to short-wavelength radiation (Ichikawa and Hideki 1982), we expect spectral composition of light to influence this process. Effects are expected to be more pronounced under green and white light (both containing short wavelengths), than under red light (comprising mainly long wavelength radiation). Insight in moth life-history under artificial night light with different spectral compositions may contribute to identification of mitigation opportunities, which are urgently needed given the rapid declines in moth populations in Western Europe, and the potential role of artificial light in this (New 2004; Conrad et al. 2006; Groenendijk and Ellis 2011; Fox 2013).

## Materials and Methods

### Study species

We used *Mamestra brassicae* (Linnaeus 1785) as a model species. This species is common in (sub-)urban areas in Western Europe, but in the Netherlands, its populations are declining (De Vlinderstichting/Werkgroep Vlinderfaunistiek 2008 and see Groenendijk and Ellis 2011), with 50–75% reduction in the number of reported catches (corrected for observation intensity) over the period 1982–2013 (Ellis et al. 2013). In Western Europe, *Mamestra brassicae* has two generations per year (Steiner and Elbert 1998; Waring and Townsend 2003). Caterpillars of the first generation develop in late spring and early summer, experiencing long days (day lengths of approximately 16 h), do not diapause and emerge as adult in late summer. The second generation caterpillars hatch late summer – early autumn. These caterpillars experience short days of approximately 11–12 h, overwinter as diapausing pupae and emerge as adults the next spring. For the present study, eggs of *M. brassicae* were obtained from a mass rearing programme at the Entomology Department of Wageningen University (see Poelman et al. (2009) for rearing details).

### Experimental set-up

**Figure 1:**
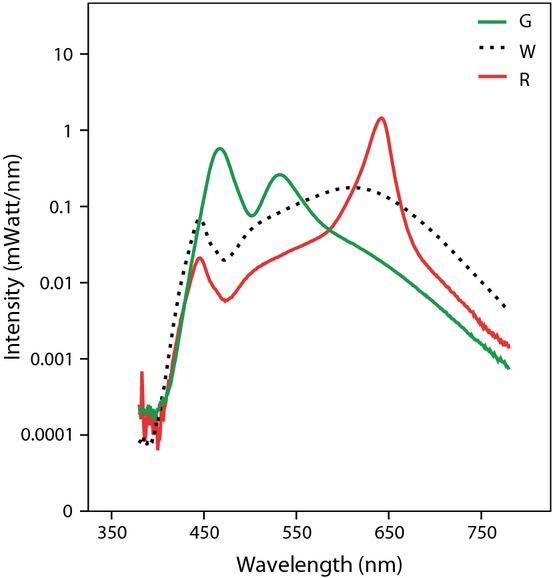
Visualisation of spectral compositions (intensities per wavelength) of the experimental lamps (G = green, W = white, R = red). Lines are averages of three lamps per type. The *Y*-axis is log scaled to enhance visibility of differences at low levels. Spectral measurements of lamps were performed at 25°C in a 2 m diameter Ulbricht sphere equipped with a Cary Varian 17D digital spectrophotometer. Lamps were allowed to stabilize for 5 min prior to measurements. A photocell was used to measure stability of the lamps and to correct spectral measurements for self-absorption by the lamp units.

In a greenhouse, we created 40 open-top compartments of 36 × 40 × 36 cm (L × W × H). These compartments were divided over 10 blocks of four compartments. The four compartments in each block were randomly assigned to one of the four artificial light treatments (green, white, red or no (dark control) artificial light at night), resulting in *n* = 10 compartments per light treatment in a randomized block design. In the compartments that were subjected to one of the artificial light treatments, we mounted LED lamps that were designed for this study by Philips Lighting (Eindhoven, the Netherlands). The spectral compositions of the red and green lamps (Fig. 1) were designed for their potential as “habitat-friendly” street lighting, aiming to minimize adverse ecological effects of artificial light by varying the amount of shorter and longer wavelengths. Lamps were made using LEDs obtained from Farnell (Utrecht, the Netherlands): each green lamp consisted of one 90 lumen cool white LED (LXML-PWC1-0090), one 23 lumen blue LED (LXML-PB01-0023) and two 80 lumen green LEDs (LXML-PM01-0080), the white lamp comprised of three 60 lumen warm white LEDs (LXML-PWW1-0060), and red lamps were made with two 70 lumen red-orange LEDs (LXM2-PH01-0070) and one 60 lumen warm white LED. LEDs were mounted in a 15 × 8 × 5 cm (L × W × H) black plastic housing. Light was mixed in a 10 cm long, 3.5 × 3.5 cm wide aluminium duct with a standard white diffusor (PPMA plate) at the end. Diffusers were adjusted to obtain light intensities of 7.0 ± 0.6 lux (measured with a LMT B360 illuminance meter [LMT, Berlin, Germany]). Although streetlight intensities are generally much higher, up to 60 lux (Gaston et al. 2012), we applied low light intensities in order to mimic light levels in the surrounding of illuminated roads. Light levels in dark control treatments were 0.04 ± 0.006 lux. Lamps were turned on at 17.00 h. (approximately 1 h before the onset of scotophase [night]) and off at 08.00 h. (approximately 1 h after the onset of photophase [day]). The day length in our experiment of approximately 11 h is similar to what second-generation *M. brassicae* caterpillars are exposed to under natural conditions. Average day temperature (measured with i-buttons (Maxim, Sunnyvale, CA) for 14 and 10 nights in block 3 and 6, respectively) was 16 ± 2°C. Average night temperature was 15 ± 1°C and was not affected by light treatment (One-Way ANOVA on average night temperatures *F*_3,3_ = 2.107, *P* = 0.278).

A random subset of 240 twelve-day old (second instar) caterpillars was selected. Each caterpillar was individually housed in a 12.5 cm high, 9.5 cm diameter white plastic pot, which was closed off with transparent plastic foil to prevent escape. Pots were randomly divided over the different compartments (i.e., six pots per compartment). Plastic foil was replaced by white insect mesh (mesh size 1.35 mm^2^, Harrod Horticultural, Lowestoft) after 23 days for better ventilation. Each caterpillar was provided with artificial diet ad libitum (see Appendix S1 for a list of ingredients) and a standardized piece of egg box for shelter. Care was taken that caterpillars were exposed to artificial light when feeding, that is, that artificial diet was not under, or in the vicinity of, the egg box.

Caterpillars were weighed at the start of the experiment, and subsequently twice a week to determine growth rate. We checked for pupation daily. Because caterpillar growth follows an exponential pattern until maximum mass is obtained, relative growth rates (RGR) until maximum mass were calculated as [ln(maximum mass) – ln(start mass)]/time (Tammaru et al. 2000). Pupal mass was recorded within 24 h after pupation, after which pupae were sexed, covered with a 2 cm layer of wood fibre and stored under a natural light-dark regime. Pots were checked daily for imago emergence, and emergence date was recorded.

### Statistical analyses

Relative growth rates until maximum caterpillar mass, maximum caterpillar mass, age at molt to pupal stage (henceforth: age at pupation), pupal mass and the duration of the pupal stage (henceforth: pupation duration) were averaged for males and females within the same compartment to avoid pseudo replication. All resulting variables fulfilled assumptions of normality and homogeneity of variances. We used a general linear model with treatment (fixed), sex (fixed), block (random) and a sex*treatment interaction as factors. In case of a significant sex*treatment interaction, analyses were performed for the two sexes separately using a general linear model with treatment (fixed) and block (random) as factors. All analyses were performed in SPSS Statistics version 20 (IBM, Amonk, NY).

Because of technical failure of one red lamp on day 17 of the experiment, data from the six caterpillars in this compartment were excluded from the analyses. In other compartments, four caterpillars died in the beginning of the experiment and data obtained from these caterpillars were also excluded from all analyses. Of the remaining 230 caterpillars, 40 caterpillars (17%) fatally failed during pupal molt. This effect was independent of light treatment (general linear model on arcsine-transformed proportions *F*_3,35_ = 1.489, *P* = 0.235). We determined sex in 37 of these 40 caterpillars, and we used data on their RGR and age at pupation for analyses. However, because pupation failure generally was accompanied by physical damage of pupae and loss of body fluids, we did not use data for analyses on pupal mass. Because we were unable to sex the other three caterpillars in which pupal molt fatally failed, data obtained from these caterpillars were excluded from both pupal mass and age at pupation analyses. Of the remaining 190 individuals, seven did not emerge from their pupae (one green, three red and three dark) and thus were excluded from analyses on pupation duration. In total, we obtained pupation durations of 183 individuals.

## Results

**Figure 2:**
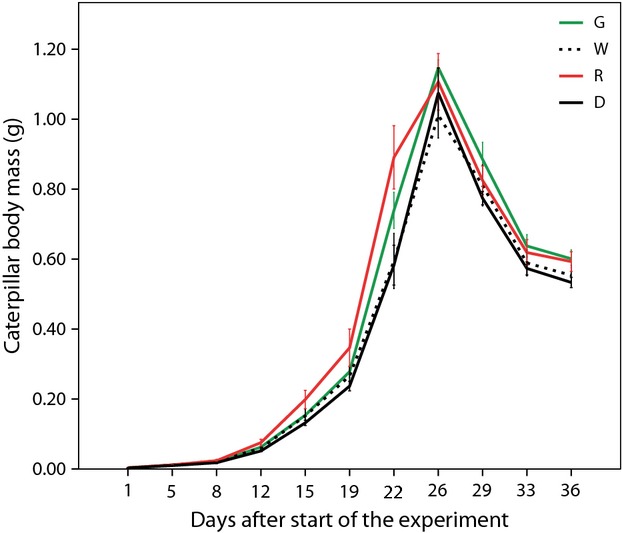
Growth curves of caterpillars (males and females combined) under the various types of artificial light at night. G = green light at night, W = white light at night, R = red light at night and D = Dark control. Error bars are 1 standard error of the mean. The peaked shape is caused by the pre-pupation inactivity period after obtaining maximum body mass.

**Figure 3:**
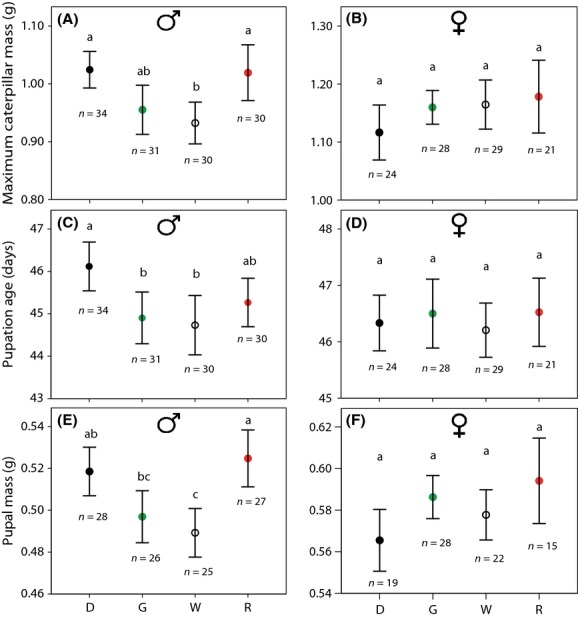
Maximum mass of male (A) and female (B) caterpillars, age at pupation of male (C) and female (D) caterpillars and pupal mass of male (E) and female (F) caterpillars subjected to different light treatments. Dots are averages, bars represent 95% confidence intervals. Different letters indicate significant differences between treatments (Tukey *α* = 0.05), and replication (*n*) is shown below the bars. Differences in replication within sexes are due to pupation failure (see Materials and Methods). D = dark control, G = green light at night, W = white light at night and R = red light at night.

Caterpillar RGR until maximum mass varied between 0.20 and 0.31 g day^−1^, and did not differ between artificial light treatments (*F*_3,57_ = 0.844, *P* = 0.476, Fig. 2). The maximum mass of the caterpillars, however, varied between 0.76 and 1.43 g, and was differentially affected by light treatment in males and females (sex*treatment *F*_3,57_ = 2.921, *P* = 0.042). When analysing sexes separately, light treatment had an effect on maximum caterpillar mass in males (*F*_3,24_ = 3.029, *P* = 0.049; Fig. 3A), but not in females (*F*_3,24_ = 0.806, *P* = 0.503; Fig. 3B). In males, mass was significantly lower for caterpillars under white light than caterpillars under red light or the dark control, whereas caterpillars under green light were intermediate (Fig. 3A).

Analyses on age at pupation and pupal mass revealed differences in artificial light effects on males and females as well (treatment*sex interactions for age at pupation [*F*_3,57_ = 2.845, *P* = 0.046] and pupal mass [*F*_3,56_ = 6.418, *P* = 0.001]). In sex-specific analyses, we found that male caterpillars that were subjected to white and green light pupated earlier than caterpillars in darkness (*F*_3,24_ = 6.260, *P* = 0.003). Caterpillars under red light were intermediate (Fig. 3C). Pupal mass in males was also affected by light treatment (*F*_3,24_ = 9.542, *P* < 0.001), with reductions of pupal mass of caterpillars subjected to white light compared to caterpillars that developed under red light or in darkness. Pupal mass of males in green light was significantly lower than that of males in red light, but not different from pupal mass of males in darkness or under white light (Fig. 3E). In females, effects on age at pupation and pupal mass were absent (*F*_3,24_ = 0.392, *P* = 0.760 and *F*_3,23_ = 1.412, *P* = 0.265, respectively; Fig. 3D,F).

**Figure 4:**
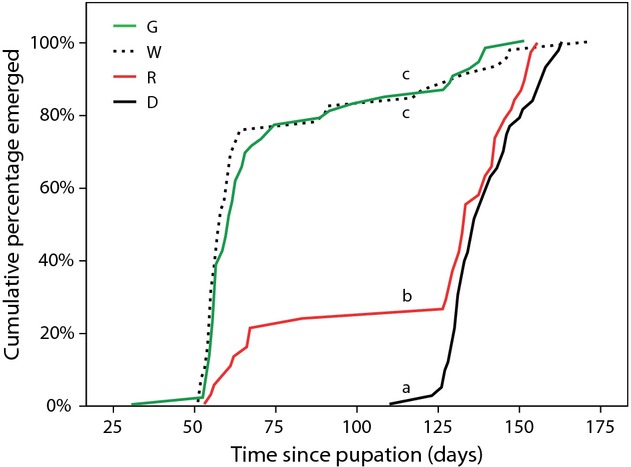
Duration of pupal stage of caterpillars that developed under the different light treatments (*n* = 44 [dark], 53 [green], 47 [white] and 39 [red]). Different letters indicate significant differences between treatments (Tukey *α* = 0.05).

Pupation duration differed greatly between light treatments (*F*_3,61_ = 26.021, *P* < 0.001; Fig. 4), irrespective of sex (sex*treatment *F*_3,61_ = 0.581, *P* = 0.630). Moths that were under green and white light as caterpillar emerged significantly earlier (after 73 (± 4) and 73 (± 5) days on average (± SE), respectively) than moths that had experienced a dark treatment as caterpillars (139 (±1.8) days, on average). Moths that had been under red light as caterpillar, had intermediate pupal development times (118 (±2) days, on average) (Fig. 4). By the time that the first moth from the dark control treatment emerged (after 110 pupal days), 85% of the moths that had been under green light as caterpillar and 83% of the moths that had been under white light as caterpillar had already emerged, whereas this was only 25% in moths that were under red light as a caterpillar. Moreover, 24%, 34% and 8% of the moths that had been as caterpillar under green, white and red light, respectively, emerged within half the time (55 days) needed for the fastest moth in darkness to emerge (Fig. 4).

## Discussion

To date, studies on the effects of artificial night lighting on moths have almost exclusively focussed on attraction of moths to sources of artificial light (Van Langevelde et al. 2011; Somers-Yeates et al. 2013). However, given the rapid declines of moth populations in Western Europe (Conrad et al. 2006; Groenendijk and Ellis 2011) and the suggested role of light pollution in this (Groenendijk and Ellis 2011; Fox 2013), there is an urgent need to move beyond attraction and study potential effects of artificial night lighting on other aspects of moth ecology. Here, we show that artificial night lighting disturbs life history and seasonal timing of moths. Male caterpillars exposed to white light reached a lower maximum caterpillar mass than males under red light and in the dark control. Furthermore, male caterpillars under green and white light pupated earlier and with a lower pupal mass than males that were under red light or in dark controls. These effects of artificial lighting on male moth life history did not result from variation in relative growth rates, as these were unaffected by artificial night lighting. Although a shorter development time (i.e., early pupation) may reduce the risk of death before reproduction (Benrey and Denno 1997; Nylin and Gotthard 1998), the concomitant reduction in mass may have negative consequences for males, such as decreased longevity, flight ability and sperm competition (Shirai 1993, 1995; Carroll 1994; Tammaru et al. 1996; Iyengar and Eisner 1999), factors that may affect a males' fitness. In females, body mass is strongly correlated to egg production (Honěk 1993) and thus directly linked to fitness. Hence, the fitness consequences of early pupation at the cost of pupal mass are more severe for females than for males (Honěk 1993; Tammaru et al. 2002). As a consequence, the trade-off between early pupation and reduced mass differs between the two sexes. This which may explain why, in contrast to males, we did not find effects of artificial night lighting on age at pupation and pupal mass in females.

The initiation of pupal diapause in moths is strongly dependent on temperature and light conditions during the larval stage (Adkisson 1966; Roditakis and Karandinos 2001; Huang et al. 2005; He et al. 2009; Pavan et al. 2010; Xiao et al. 2010; Xu et al. 2014). We found very large differences in the duration of the pupal stage, with a vast majority of moths subjected to green and white artificial light as larva emerging earlier from the pupa than moths in darkness (Fig. 4) for both sexes. These differences are likely a result of suppression of diapause initiation by exposure to green and white light at night in the larval stage. Low intensities of artificial light have previously been shown to interfere with natural light-dark cycles as an information source about timing and location in a variety of other species groups (Gaston et al. 2013). Indeed, the low levels of artificial light used in our experiment are sufficient to interfere with day length as an environmental cue for seasonal timing of pupal diapause in moths. Under natural conditions, the inhibition of pupal diapause by artificial light at night can cause de-synchronisation of imago emergence, leading to mismatches with favourable seasons (Adkisson 1966) and thus high mortality as non-diapausing *M. brassicae* pupae are unlikely to survive the winter. Although, moths that were under red light also emerged on average earlier than moths in darkness, the pattern in emergence resembled that of moths in darkness (Fig. 4).

Spectral alterations have been suggested to provide a tool for mitigation of negative effects of artificial light on ecosystems (Van Langevelde et al. 2011; Gaston et al. 2012; Fox 2013). Our results show that negative effects of artificial light on moth life history and seasonal timing can be reduced by changing spectral compositions. Overall, compared to white and green light, the effects of red light at night on moth life history and seasonal timing were less strong, or even absent. This can be explained by a low sensitivity of caterpillar stemmata to longer wavelengths such as red light (Ichikawa and Hideki 1982).

We conclude that low levels of artificial light at night affect key life history traits in moths and interfere with light as a cue for seasonal timing in moths, thereby disrupting the initiation of diapause. These effects can have important negative fitness consequences, which add up to other adverse effects of artificial light on moths such as attraction (Van Langevelde et al. 2011; Somers-Yeates et al. 2013) and the associated higher predation levels (Rydell 1992; Svensson and Rydell 1998; Acharya and Fenton 1999), and thus are likely to contribute to the observed widespread declines in moth populations in Western Europe (Conrad et al. 2006; Groenendijk and Ellis 2011; Fox et al. 2013). Furthermore, we show that the application of light that is poor in short-wavelength radiation (i.e., red light) can be used to at least partly mitigate negative effects of nocturnal illumination on moth life history and seasonal timing.

## Supporting Information

**Appendix S1.** Ingredients of the artificial diet.
