# Supplementary material for: Artificial light at night causes diapause inhibition and sex-specific life history changes in a moth
Source: Ecol Evol. 2014 Apr 25;4(11):2082–9. doi: 10.1002/ece3.1090 (PMC4201423; doi:10.1002/ece3.1090)
Supplement: Supplementary file 1 — Appendix S1. Ingredients of the artificial diet. [file ece30004-2082-sd1.doc]

**Appendix S1.** Ingredients of artificial diet for *Mamestra brassicae* caterpillars

2 L water

70g agar (Sigma-Adlrich Chemie GmbH, Steinheim, Germany) in 0.5L water

350g polenta (Windkorenmolen “de Vlijt”, Wageningen, the Netherlands)

125g yeast flakes (Natudis, Harderwijk, the Netherlands)

125g wheat germ (Natudis, Harderwijk, the Netherlands)

5g sorbic acid (Carl Roth GmbH, Karlsruhe, Germany)

4g Methyl 4-hydroxylbenzoate, 99% (Acros Organics, Geel, Belgium)

20g ascorbic acid (Hinmeijer Chemic V.O.F., Haarlem, the Netherlands)

0.25 Streptomycin sulphate (Sigma-Adlrich Chemie GmbH, Steinheim, Germany)
